# Exploring interpersonal neural synchrony in coach-athlete interactions: insights from naturalistic EEG hyperscanning in competitive tennis

**DOI:** 10.3389/fspor.2026.1751291

**Published:** 2026-02-12

**Authors:** Davide Crivelli, Michela Balconi

**Affiliations:** 1 International research center for Cognitive Applied Neuroscience (IrcCAN), Università Cattolica del Sacro Cuore, Milan, Italy; 2 Faculty of Psychology, Università Cattolica del Sacro Cuore, Milan, Italy

**Keywords:** coach-athlete communication, EEG, hyperscanning, naturalistic interaction, real-time feedback, sport

## Abstract

Effective communication between a coach and athlete is fundamental in sports, influencing the athlete's physical and psychological well-being, performance, and long-term development. Key principles of effective coach-athlete (Co-At) interaction include optimized communication skills, empathic resonance, mirroring, and syntonization, which enhance understanding and relational attunement. This study aimed to deepen our understanding of these dynamics by investigating Interpersonal Neural Synchrony (INS) using a naturalistic EEG hyperscanning paradigm in tennis. Namely, starting from field recording of naturalistic feedback exchanges in 11 Co-At dyads, we computed Dyadic Dissimilarity Metrics (DDM) in structured (i.e., interactive review sessions) and unstructured (i.e., interactions during a match) settings. Findings highlighted four key points. Structured compared to unstructured interactions were generally perceived as more effective, as well as – by athletes – more affectively engaging. Lower DDM values, indicating greater INS, were generally observed during structured feedback. Significant neural alignment was noted in the right prefrontal, bilateral centro-temporal, and left parieto-occipital regions during structured interactions. And, finally, regional differences in DDM emerged, with the right prefrontal region showing the highest INS in structured settings and left centro-temporal regions showing the lowest in unstructured ones. These findings underscore the importance of structured communication for fostering neural patterns similarity and optimizing coaching feedback.

## 1 Introduction

Effective communication between a coach and athlete is fundamental in the context of sports, impacting not only the athlete's physical and psychological well-being but also their performance and long-term development. This unique interaction, characterized by a high degree of immediacy, adaptability, and feedback, exemplifies a form of interpersonal communication central to fostering athletic growth and optimizing skills (1). Coaches engage with athletes in a continuous exchange of verbal and non-verbal cues, delivering feedback, encouragement, and correction aimed at refining both immediate performance and facilitating the longer-term acquisition of complex motor and cognitive skills. This communication is not merely transactional but relational, involving a rich interplay of empathy, emotional regulation, and situational awareness.

As a distinct instance of interpersonal relationship, the coach-athlete (Co-At) interaction necessitates attention to the communicative dynamics that drive an athlete's motivation, resilience, and emotional stability. Unlike other forms of interaction, communication in this relationship is marked by the alignment of goals and an integrated focus on both immediate tactical adjustments and broader developmental objectives. Recent work has emphasized the role of augmented verbal feedback in enhancing motor performance and skill acquisition in practice and competition settings, highlighting the importance of delivering precise, constructive, and adaptive feedback (2, 3). This interpersonal dimension situates Co-At communication within the broader domain of relationship studies, emphasizing the need for a consistent and flexible approach to feedback that supports skill retention, positive affect, and overall satisfaction (1).

Central to effective Co-At interaction are the principles of optimized communication skills, empathic resonance, mirroring, and syntonization, which help foster understanding and relational attunement between coach and athlete (4–10). As noted above, communication skills serve as a critical component in guiding athlete development, enhancing motivation, and facilitating optimal performance, especially through clear, adaptive, and responsive feedback (11–13). Empathy enables a coach to tune into an athlete's cognitive and emotional states, facilitating an adaptive communication style that meets the athlete's evolving needs and learning stage (14, 15). This empathic resonance is essential not only for enhancing motivation but also for recognizing subtle shifts in an athlete's focus, confidence, or physical state, allowing for real-time adjustments in coaching strategy. Mirroring, in turn, supports this process by allowing the coach to reflect the athlete's body language, expressions, and energy levels, thus creating a reinforcing feedback loop that strengthens the athlete's sense of being understood and supported (16, 17).

The concept of syntonization further builds on these dynamics, involving a synchronized exchange of cues and responses between coach and athlete. This process has been explored through frameworks such as two-person neuroscience, which investigates the neural underpinnings of interpersonal synchronization and shared intentionality (18, 19). In practical terms, syntonization may be a key enabler in establishing an empowering relational dynamic between coach and athlete, enhancing the clarity and relevance of instructional cues and allowing for smoother communication in high-stakes or pressure-filled situations. This inter-brain synchronization is observed through methods like hyperscanning, which has shown that synchrony in brain activity can predict the efficacy of social interactions and shared tasks (20–23). Going down to specifics, hyperscanning requires the simultaneous recording of behavioural and physiological activities from multiple individuals engaged in social interactions or a shared task, as well as the integrated analysis of such activities considering dyads or groups of individuals – and not the single individuals – as observational units (21, 24–27). Such investigation paradigm holds a valuable potential in studying complex relational dynamics such as those between coaches and athletes, as pointed out in other applied research contexts (8, 28–30). Yet, such potential is still largely unexplored in the field of sport science and practice.

Within the framework of two-person neuroscience, interpersonal neural synchrony (INS) refers to the systematic alignment or similarity of neural activity patterns between interacting individuals over time. Importantly, INS is not conceived as a direct neural correlate of shared representations or identical mental states, but rather as a relational and context-dependent marker reflecting the degree to which interacting agents are jointly engaged, coordinated, or attuned within a given interactional setting (24, 26, 31, 32). Contemporary accounts emphasize that INS emerges from the dynamic coupling of perception, action, and communication processes, and is shaped by task structure, role asymmetry, interaction goals, and environmental constraints, rather than reflecting a unitary or static phenomenon. In the Co-At context, hyperscanning may reveal critical patterns in the timing and coordination of neural responses that correlate with effective syntonization between interacting individuals, especially in situations requiring continuous feedback and quick adjustment of communication exchanges. For example, INS has been linked to better alignment in task objectives, faster response times, and enhanced mutual understanding in real-time interactions (21, 33). Furthermore, research on INS has demonstrated that synchronized brain activity between inter-agents can enhance communicative efficacy, reinforcing shared attention and intentionality in a way that promotes a collaborative and adaptive learning environment (e.g., 34, 35). This insight is invaluable for high-pressure environments such as competitive sports, where feedback exchanges occur under dynamic and challenging conditions.

In training sessions, hyperscanning may elucidate the neural mechanisms underlying feedback processing and adaptation to coaching cues, potentially revealing how athletes' neural responses evolve across sessions as they internalize the coach's guidance. Evidence suggests that aligned neural activity can reflect the quality and depth of engagement during training, as well as the degree of attunement between a coach and a coachee (e.g., 36, 37). Also, in competitive settings where coaches and athletes must often adapt to split-second changes, hyperscanning can help pinpoint moments where feedback either enhances or disrupts the synchrony necessary for optimal performance. Such applications can deepen our understanding of how stress, excitement, and other emotional states influence the brain's receptiveness to coaching feedback, offering pathways to refine feedback timing and style. Feedback plays a central role in guiding athlete development, and hyperscanning techniques hold the potential to provide greater insight into how feedback can be tailored to maximize its effectiveness. Studies using hyperscanning could significantly enhance our understanding of the specific neural correlates of feedback, identifying the optimal conditions under which feedback fosters neural alignment and enhances learning outcomes.

Further, hyperscanning studies have revealed that communication efficacy in feedback-laden scenarios is associated with anticipatory brain responses in the listener, with respect to the speaker's brain activity, in successful exchanges (38, 39). This suggests that athletes in sync with their coach may intuitively anticipate instructions or corrections, aligning their neural activity with the coach's guidance even before explicit feedback is given. This phenomenon, observed across studies of cooperative tasks (31, 40–43), underscores the role of anticipation and joint attention in fostering successful Co-At relationships and improving training outcomes.

Focusing even more on naturalistic contexts for sport practice, EEG hyperscanning might offer a way to assess social attunement in real time, tracking shifts in attention and working memory engagement as they occur during feedback exchange. By mapping Co-At attunement in real-life social dynamics, this approach might provide a valuable neurophysiological perspective on how effective communication and feedback can enhance athletic performance. Alpha power (8–12 Hz), in particular, has been widely used as a marker of task-related activation in EEG hyperscanning studies, with alpha suppression commonly observed during social interactions and cooperative tasks as individuals engage in shared processing. Suppression of alpha rhythms has been associated with the mirror neuron system (MNS), which underpins imitation and empathy, essential components of effective social coordination (4, 44–47). The presence of alpha suppression, especially in the right central and sensorimotor regions - often linked to the mu rhythm or to the phi complex - has been identified as a marker for social coordination, suggesting the relevance of alpha rhythms in facilitating communication across social contexts (20, 48, 49; but see also 50).

In Co-At interactions, the use of hyperscanning to measure alpha power might provide insight into moments of neural alignment during both structured and unstructured feedback scenarios. Studies on inter-brain synchronization have demonstrated that alpha suppression patterns can vary based on the type of interaction. For example, joint attention tasks often show left centro-parietal alpha suppression (51), while verbal interactions and turn-taking activities, such as those involving speech rhythm coordination, can show suppression in bilateral centro-parietal regions and left temporal region (52, 53). In these contexts, alpha suppression has been linked to shared short-term memory demands and attentional alignment, crucial for accurately tracking and responding to a partner's actions (54, 55). Further evidence supports the utility of alpha synchrony in tasks involving verbal interaction and alternating speech, where synchronization in such frequency band contributes to successful information exchange and turn-taking between partners (38, 39, 56). Notably, inter-brain phase synchronization in EEG has been shown to correlate strongly with collective performance and shared cognitive processing, potentially serving as a more reliable index of engagement and coordination of neural responses supporting interaction than self-report measures alone (31, 42).

In parallel with the growing interest in ecological and second-person neuroscience, EEG hyperscanning has increasingly been applied to movement-based and action-oriented contexts, including joint motor coordination, cooperative tasks, and embodied interaction. Studies have shown that inter-brain synchrony emerges during coordinated movements, joint action, and sensorimotor coupling, and that such synchrony is modulated by task demands, role differentiation, and interaction structure (e.g., 20, 31, 41, 49). More recently, hyperscanning approaches have been used to investigate motor expertise, action observation, and coordination in ecologically valid settings, bridging social neuroscience with movement science and applied domains (42, 57, 58). For example, Liu and colleagues (59) applied EEG hyperscanning in an ecological, sport-related context using a motion-sensing tennis game to investigate the neural correlates of cooperation and competition during interactive motor behaviour. By analysing inter-brain amplitude correlations and phase alignment, the authors showed that cooperation and competition are associated with distinct inter-brain coupling patterns, particularly in the theta, alpha, and beta frequency bands. Importantly, their findings highlighted the sensitivity of amplitude-based inter-brain measures to differences in social interaction modes during coordinated motor activity. And again, Tamburro and colleagues (60, 61) tried to extend EEG hyperscanning to real sport practice by examining inter-brain dynamics during cooperative and competitive table-tennis interactions in fully ecological settings, with a focus on methodological and analytical frameworks for investigating dyadic joint motor action.

However, despite this emerging body of work, applications of EEG hyperscanning within sport science remain scarce, and – to the best of our knowledge – no previous study has directly investigated interpersonal neural synchrony during real coach–athlete interactions in an authentic training or competitive context. The present study builds on movement- and interaction-based hyperscanning research by extending it to a core applied sport setting, focusing on the neural correlates of communication and feedback exchanges between coaches and athletes under naturalistic conditions.

To sum up, given the crucial role of coaches in the development and success of athletes – and the significant impact this relationship has on sports performance - systematically exploring the effects of Co-At communicative interactions is a priority of both academic and practical significance. This study aimed to investigate the neurofunctional correlates of these interactions, using tennis as a testbed discipline. Specifically, we focused on EEG-derived INS during both structured and unstructured feedback exchanges, to better understand how communication dynamics between coaches and athletes in primary manifestations of Co-At interaction influence their syntonization and social attunement. The inclusion of both structured and unstructured interaction settings was intentional, as they exemplify two predominant categories of feedback exchanges between coaches and athletes. Structured contexts, such as coaching sessions during training or post-competition reviews, provide a clear framework for feedback delivery. In contrast, unstructured contexts, like real-time exchanges during a match or moments when the athlete is performing, occur within a fluid and dynamic environment where the timing and location of communication are not pre-determined. By examining both types of settings, we tried to capture a broader spectrum of communicative scenarios that shape the coach-athlete relationship.

The secondary objective was to investigate the feasibility and informativity of an integrated neuroassessment protocol dedicated to the quality of Co-At communication, based on a naturalistic EEG hyperscanning paradigm. This innovative method allows for examining and measuring the electrophysiological activations and behaviour of multiple individuals engaged in communicative exchanges in real-life environment and during naturalistic interaction, enabling joint analysis of the data collected. The use of hyperscanning during naturalistic interactions, indeed, provides an innovative opportunity to explore the relational dynamics between coaches and athletes as they emerge and develop in everyday situations, allowing for a deeper understanding of the neurofunctional foundations underlying such complex form of inter-personal relationship. Accordingly, in the present study INS is interpreted as an indirect and context-sensitive index of interpersonal attunement, whose meaning must be understood in relation to interaction structure and communicative demands.

Notwithstanding the exploratory nature of the pilot study, available literature allowed us to formulate a few hypotheses. Namely, we expected that participants would have perceived communication as more effective during a structured feedback exchange then in an unstructured situation. The clear framework for feedback delivery, the protection from external nuisance or intrusive interference, and expectable nature of bidirectional communication exchanges would likely foster perceived efficacy.

In addition, given the role of alpha activity in social understanding and coordination processes, we posited that such component of EEG activity might have been a good target for computation of INS metrics and might have provided insights on neural syntonization during both structured and unstructured feedback scenarios. Then, given the role of alpha modulations and synchronization in successful information exchange and turn-taking between partners, we have specifically hypothesized that, comparing a structured session to unstructured feedback exchanges during a match, the former condition would have fostered more pronounced alpha-based INS metrics between coach and athlete than the latter, indicating a stronger synchronization of brain activity between the two participants. Finally, we have nonetheless hypothesized that alpha-based INS markers would have emerged over frontal regions even during unstructured feedback moments, despite the absence of structured bidirectional exchanges, mirroring the influence on athlete's cognitive and top-down regulatory processes of coach's direct feedback during play.

## 2 Materials and methods

### 2.1 Sample

The study sample included 11 competitive Series C tennis players (5 women and 6 men, M_age_ = 23.7, SD_age_ = 4.3) affiliated with a tennis club located in Milan and five certified tennis coaches (3 men and 2 women, M_age_ = 44.0, SD_age_ = 13.4) who regularly worked with the athletes, forming a total of 11 unique coach-athlete dyads. Some coaches were paired with more than one athlete, resulting in partial nesting of dyads within coaches (each coach was paired with the two or three athletes he/she personally trained). This dyads structure was taken into account in the statistical analysis, as described below. Athletes were selected based on their advanced skill level in tennis and regularly participated in training sessions and competitive matches alongside their respective coaches.

Participation was voluntary (no monetary compensation was provided), and both athletes and coaches received detailed information regarding the study and the protocols involved. Consecutive sampling was used to enrol participants. Exclusion criteria included any concurrent psychiatric or neurological disorders, clinical signs of anxiety, depression, or stress, having suffered from a sport-related injury in the past 6 months, and chronic pain. Inclusion criteria was: being over 18-yo; being a competitive tennis player at least at Series C level or a certified tennis coach; normal or corrected-to-normal vision and hearing.

Written informed consent was obtained from all participants, and the study adhered to the principles and recommendations of the Declaration of Helsinki (2013) and of both national and international regulations in terms of data protection and privacy (GDPR - Reg. UE 2016/679). The research protocol received approval from the Ethics Committee of the Department of Psychology, Università Cattolica del Sacro Cuore.

### 2.2 Procedure

The study procedure was designed to capture Co-At naturalistic interactions during coaching sessions and tennis matches, allowing for the assessment of neurofunctional correlates of interpersonal syntonization in both structured and unstructured real-life social exchanges connoted by particular relevance and saliency for both inter-agents.

The study, then, included two hyperscanning sessions – namely, the structured vs. unstructured feedback condition. Before each hyperscanning session, a brief resting-state baseline was recorded with eyes open for both participants, while seated and instructed to remain still (duration: 2 min). This baseline was used exclusively for within-subject normalization of task-related EEG power and was not intended as a control condition. In the structured feedback condition, each coach-athlete dyad participated in a 10-minute interactive coaching session, with no specific restrictions on content or interaction structure. Data were collected in a dedicated area on the tennis fields provided by the tennis club. Topics emerging from such interactions included: feelings, thoughts, and sensations experienced during the last match; successful and unsuccessful hits and their antecedents in terms of both movement and thoughts; felt attitude towards the match; strong-points and weak-points that could become target for further training. The unstructured feedback condition, instead, took place during a short training match (namely, a 10-point tie-break) lasting, on average, 20 min. In such matches, each athlete face a competitor with similar experience, so to keep the challenge and engagement levels comparable to those during a cull competitive match. During the match, typically at the end of each point, the coach shared with the athlete feedbacks and comments, and the athlete had the opportunity to reply while trying to keep timings as similar as possible to those of a real competition. Data were continuously collected during the match and during the feedback exchanges from both coaches and athletes. The structured and unstructured conditions were defined *a priori* based on the presence (structured) or absence (unstructured) of an organized interactional framework, including turn-taking expectations, temporal boundaries, and communicative focus. In both conditions, Co-At interactions were videorecorded.

After each hyperscanning session, participants were asked to complete a brief questionnaire to evaluate the quality and perceived effectiveness of the Co-At interaction. The questionnaire was constituted by four items investigating: i) perceived communication efficacy [“*I perceived the interactions I had with the (athlete/coach) as effective*”]; ii) pleasantness of the interactions [“*I perceived the interactions I had with the (athlete/coach) as pleasant*”]; iii) affective engagement in the communication exchanges [“*I perceived the interactions I had with the (athlete/coach) as affectively engaging*”]; iv) cognitive effort in managing the interactions [“*I perceived the interactions with the (athlete/coach) as cognitively effortful to manage*”]. Participants answered the items using a 5-point Likert scale (1 = Strongly disagree; 2 = Somewhat disagree; 3 = Neither agree nor disagree; 4 = Somewhat agree; 5 = Strongly agree). These subjective reports were included so to complement the analysis of physiological corelates on INS and offer a nuanced view of Co-At interactions by combining objective neurophysiological data with subjective perceptions.

Data collection was always planned during the same afternoon, in correspondence to one of athletes' training and practice sessions, with a washout period between sessions to reduce fatigue and allow participants to adapt comfortably to the experimental setup. We opted for such strict planning in order to try and keep recording conditions as similar as possible across data collection and to minimize the risk of potential intrusion of situational or environmental confounding variables. Also, the order of the conditions (i.e., structured vs. unstructured) was counterbalanced across dyads so to prevent potential confounds in data interpretation due to time-order or fatigue effects. As a final note on general study methodology, we chose to plan data collection in correspondence to participants' training sessions and using the tennis fields as the recording environment in order to keep the actual contexts of Co-At interactions as natural and realistic as possible, in the attempt to ecologically capture real-life communication dynamics. For the same reason, the dyads were constituted by athletes and their personal coach, and were not randomly generated. By that, we once again wanted to facilitate the emergence of realistic social exchanges, each connoted and enriched by specific communication and relational customs and habits.

### 2.3 EEG recording and reduction

For brain activity recording during Co-At interactions, wireless EEG devices (EpocX, Emotiv Inc., San Francisco, CA, USA) equipped with 14 semi-dry sensors was used, chosen for their portability, ease-of-use, and reliability even in real-life contexts and in dynamic sports environments. The recording montage included the following sites: AF3, F7, F3, FC5, T7, P7, O1, O2, P8, T8, FC6, F4, F8, AF4. Two CMS/DRL references was placed in correspondence to the left/right mastoid process. Before each recording, the sensors were dampened with a saline solution, and athletes' hair was carefully moved aside to optimize scalp contact. Then, EEG devices were covered and stabilized using tubular gauze to maintain electrode placement and equipment stability even during the match session. This preparation ensured safer recordings suitable for hyperscanning in both structured and unstructured settings.

Signals were sampled at 2048Hz and then downsampled when extracted to 256 Hz, using 0.16–43 Hz bandwidth and a 50 Hz digital notch filter. Signal quality were constantly monitored during each hyperscanning session. Beginning and end of communication exchanges, as well as turn-taking, was marked in the EEG recording during data collection in the structured and unstructured conditions. For both conditions, EEG recordings were temporally aligned offline with video recordings of Co-At interactions so to align timing of collected data, verify event markers and set final synchronized markers signalling speaking turns in the communication exchanges. This synchronization procedure ensured correspondence between neural data and interactional events across both participants.

The collected EEG data were analysed offline. Given the ecological nature of the recordings, particular care was taken to mitigate movement-related artefacts through both procedural (sensor stabilization and contact, continuous signal quality monitoring) and offline preprocessing steps. Biosignals processing included a 1–40 Hz bandpass filter with a 48 dB/oct slope. Signals were then segmented by condition (baseline - BL, structured interaction - STR, and unstructured interaction - UNS) and speaking turn (athlete – ATH, coach – COA). Although the overall duration of the structured and unstructured recording sessions differed (approximately 10 and 20 min, respectively), the interaction segments included in the EEG analyses were duration-matched across conditions. Specifically, only stationary segments corresponding to coach–athlete feedback exchanges were selected for analysis. In the unstructured condition, these segments occurred during the brief pauses between points, when coaches typically provide feedback and athletes are stationary. Data quantity and signal quality were systematically controlled prior to power spectral density extraction, and only artifact-free segments were retained for subsequent analyses. Indeed, besides stringent filtering, the data were further inspected to identify and remove any further potential ocular, motor, or movement artifacts. Artifact-free segments were processed using Fast Fourier Transformation (FFT) to compute power spectra from the oscillatory activity, which were then averaged. Mean Power Spectral Density (PSD) values for alpha band was extracted for each experimental condition. Overall, the preprocessing pipeline was designed to balance signal preservation with artefact attenuation, in keeping with principles of good-practice for mobile and naturalistic EEG hyperscanning.

Finally, task-related PSD values for the specified frequency bands in the different experimental conditions (structured interaction, unstructured interaction; athlete vs. coach speaking turn) were calculated relative to individual resting-state EEG metrics collected during specific eyes-open baselines, using the following computation:

$$TR_{\mathrm{PSD}}=\;[\ln(\mathrm{PS}D_{\mathrm{cond}})\;-\;\ln(\mathrm{PS}D_{\mathrm{bl}})]\;/\;\ln(\mathrm{PS}D_{\mathrm{bl}})$$

where TR_PSD_ represents task-related variations in EEG PSD values for each electrode, ln(PSD_cond_) represents ln-transformed PSD values during each specific condition for each electrode, and ln(PSD_bl_) represents ln-transformed PSD values in eyes-open baseline for each electrode.

From these data, response profiles were calculated for specific regions of interest (RoIs): left prefrontal area (electrode sites: AF3, F7, F3), right prefrontal area (electrode sites: AF4, F8, F4), left centro-temporal area (electrode sites: FC5, T7), right centro-temporal area (electrode sites: FC6, T8), left parieto-occipital area (electrode sites: P7, O1), and right parieto-occipital area (electrode sites: P8, O2). Then, to investigate levels of interpersonal synchrony using INS metrics, task-related responses from the athlete and coach in each RoI were used to calculate dyadic dissimilarity metrics (DDM), quantified as the Euclidean distance between the athlete's and coach's TR_PSD_ values in each condition and RoI. As such values are scalar, the Euclidean distance corresponds to absolute differences in task-related variations of alpha power between the two inter-agents, reflecting the degree of similarity in activation magnitude. This metric, then, provides an amplitude-based index of similarity between inter-agents' neural responses, with lower values indicating closer neural activation patterns in the dyad (signalling higher INS), while higher DDM values indicate greater dissimilarity in inter-agents' neural activities. DDM was selected because it is well suited for naturalistic and mobile EEG hyperscanning contexts, as it does not rely on precise phase locking or stationarity assumptions and is comparatively robust to residual noise and movement-related variability. Moreover, distance-based metrics allow for a straightforward interpretation of inter-brain similarity while preserving sensitivity to regional and contextual modulation of neural responses. DDM in the RoIs for each experimental condition were used in inter-brain statistical analyses.

### 2.4 Statistical analysis

The data analysis plan included two main steps. Firstly, athletes' and coaches' responses to the brief questionnaire on the quality and perceived effectiveness of the Co-At interaction were analysed via ANOVA models. Secondly, INS data quantified as DDM metrics for each RoI and condition were analysed via linear mixed-effects modelling. Linear mixed-effects modelling was adopted to account for the hierarchical and non-independent structure of the data, with repeated interaction turns nested within coach-athlete dyads. Statistical tests and models have been run and checked using *jamovi* (version 2.4; 62) and, specifically for mixed-effects models, the *GAMLj3* module (version 3.1.4; 63).

Going down to specifics, self-report data concerning perceived communication efficacy, pleasantness of the interactions, affective engagement during communication exchanges, and cognitive effort in managing the interactions were analysed through mixed-design ANOVA models including Condition (2 levels: structured feedback condition – STR vs. unstructured feedback condition – UNS) as within-subject factor and Role (2 levels: athlete – ATH vs. coach – COA) as between-subject factor. Tukey tests were used in pair-wise comparisons to further explore statistically significant simple effects while accounting for multiple comparisons bias. Partial eta squared (*η_p_^2^*) was computed to assess the size of significant effects. Effect size were deemed as small when *η_p_^2^* values was between 0.01 and 0.06, medium when *η_p_^2^* values was between 0.06 and 0.14, and large when *η_p_^2^* values was greater than 0.14, in agreement with Cohen's norms (64). Threshold for statistical significance was set to *α* = 0.05.

Then, differences in DDM values across RoIs and conditions have, instead, been tested by means of a linear mixed-effects model. Condition (2 levels: structured feedback condition – STR vs. unstructured feedback condition – UNS), Speaker (2 levels: athlete – ATH vs. coach – COA), Area (pre-frontal – PF; centro-temporal – CT, parieto-occipital – PO), and Side (left – L vs. right – R) has been included as fixed effect in the model. Dyad was included as a random effect to control for within-couple dependency, as well as for between-couple variability and subjective differences between Co-At couples. Given the limited number of coaches (*n* = 5), coach-level clustering could not be reliably estimated as a separate random effect and was therefore not modelled explicitly. Turn order (the sequential number identifying the progressive communication turns taken by athletes or coaches within each Condition) has been included in the model as covariate to account for potential confounds concerning sequence or time-related effects during interaction. An autoregressive first-order structure was applied to model residuals in order to address potential temporal autocorrelation across successive turns, which is expected in continuous and naturalistic interaction settings. This modelling strategy was chosen to maximize statistical robustness while preserving sensitivity to condition- and region-specific effects. Model assumptions were assessed through inspection of residual distributions and diagnostic plots prior to inference. The significance of terms for the fixed effect has been assessed by conditional *F*-tests; we will then report relative F and *p*-values of the Type III Sum of Squares computations. Statistical significance threshold was set to *α* = 0.05. Pair-wise comparisons used to explore simple effects included Bonferroni correction adjusting for multiple testing biases. Cohen's *d-*values were calculated and reported as a measure of effect size for statistically significant pair-wise comparisons. Effect size were deemed as small when *d*-values was between 0.20 and 0.50, medium when *d*-values was between 0.50 and 0.80, and large when *d*-values was greater than 0.80, in agreement with Cohen's norms (64).

## 3 Results

**Figure 1:**
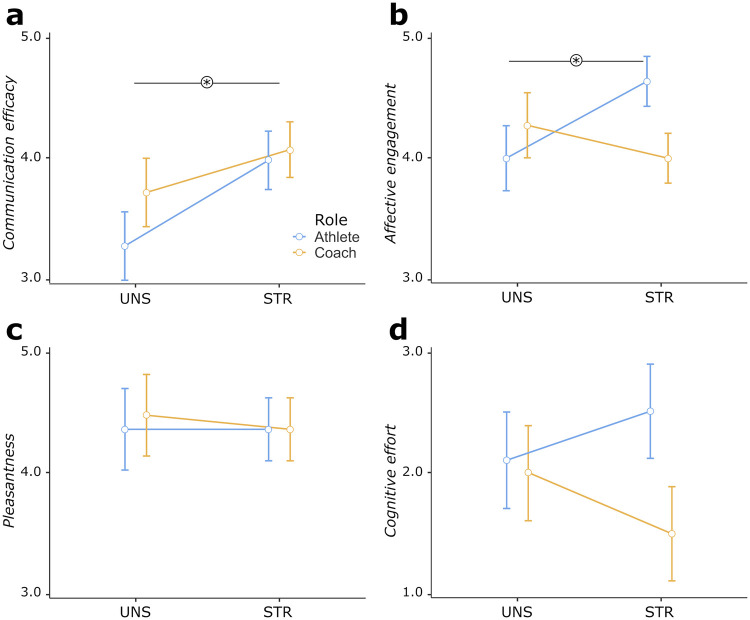
Comparison between athletes’ and coaches’ self-reported data in structured (STR) and unstructured (UNS) interaction conditions: **(a)** perceived communication efficacy; **(b)** pleasantness of the interactions; **(c)** affective engagement during the communication exchanges; and **(d)** cognitive effort in managing the interactions. Bars represent ±1 SE from the mean. Asterisks mark statistically significant comparisons (*p* < .05).

The ANOVA model on perceived communication efficacy highlighted a statistically main interaction effect of Condition [*F*(1,20) = 6.990, *p* = 0.016, *η_p_^2^* = 0.26], with generally greater efficacy ratings during STR than the UNS condition [EM_STR_ = 4.05, 95% CI (3.75, 4.34); EM_UNS_ = 3.50, 95% CI (3.12, 3.88); see Figure 1a].

The ANOVA model on affective engagement ratings highlighted a statistically significant Condition*Role interaction effect [*F*(1,20) = 8.470, *p* = 0.009, *η_p_^2^* = 0.30]. Pair-wise comparisons revealed significantly higher ratings scores for the STR than the UNS condition, specifically among athletes [*p* = 0.042; EM_ATH−STR_ = 4.64, 95% CI (4.28, 5.00); EM_ATH−UNS_ = 4.00, 95% CI (3.51, 4.49); see Figure 1b].

No other ANOVA model highlighted statistically significant main or interaction effects (see Figures 1c,d).

The analysis of DDM values for the alpha band showed significant main effects for the Condition [*F*(1,2131) = 122.535, *p* < 0.001], Area [*F*(1,2131) = 11.669, *p* < 0.001], and Side [*F*(1,2131) = 3.852, *p* = 0.050] factors. In addition, the model highlighted a significant two-way interaction between Condition and Area [*F*(2,2131) = 6.178, *p* = 0.002] and a three-way interaction between Condition, Area, and Side [*F*(2,2131) = 5.607, *p* = 0.004].

Namely, DDM values were lower in the STR than in the UNS condition [EM_STR_ = 0.89, 95% CI (0.54, 1.25); EM_UNS_ = 1.71, 95% CI (1.34, 2.07)]. Also, DDM values were globally lower in PF than CT (*p* = 0.002) and PO (*p* < 0.001) areas [EM_PF_ = 1.03, 95% CI (0.65, 1.41); EM_CT_ = 1.37, 95% CI (0.99, 1.74); EM_PO_ = 1.50, 95% CI (1.13, 1.88)], and in right than left side [EM_R_ = 1.22, 95% CI (0.85, 1.59); EM_L_ = 1.38, 95% CI (1.02, 1.75)].

**Figure 2:**
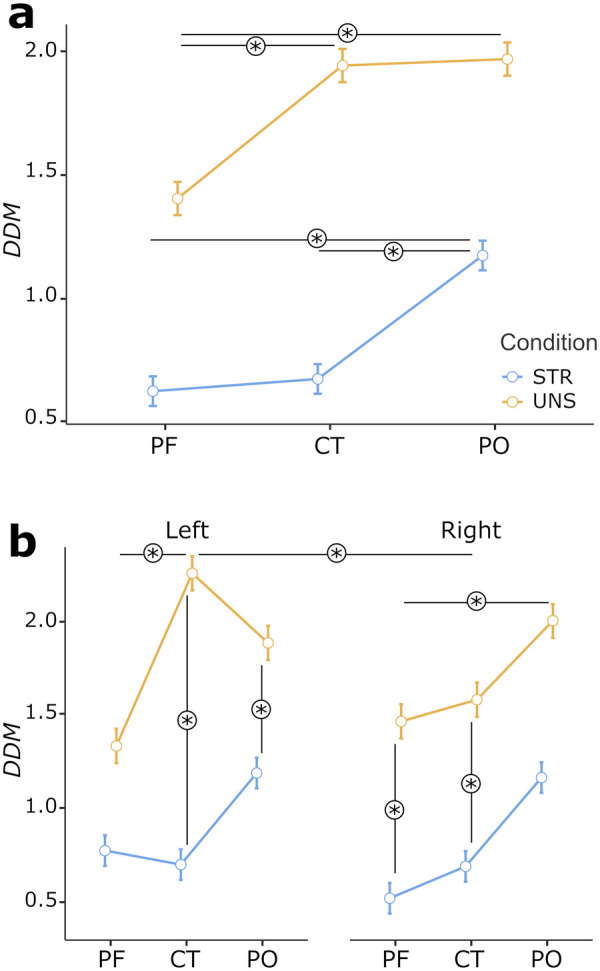
**(a)** comparison of dyadic dissimilarity metrics (DDM) in structured (STR) and unstructured (UNS) interaction conditions across prefrontal (PF), central-temporal (CT), and parietal-occipital (PO) regions (two-way interaction effect). **(b)** Comparison of DDM in STR and UNS interaction conditions across left vs right PF, CT, and PO regions (three-way interaction effect). Bars represent ±1 SE from the mean. Asterisks mark statistically significant comparisons (*p* < .05).

In addition, pair-wise comparisons highlighted that DDM values – in the STR condition – were significantly lower in correspondence to PF (*p* < 0.001) and CT (*p* = 0.004) than the PO [EM_STR−PF_ = 0.71, 95% CI (0.32, 1.09); EM_STR−CT_ = 0.78, 95% CI (0.39, 1.17); EM_STR−PO_ = 1.20, 95% CI (0.81, 1.59)], while - in the UNS condition - they were significantly lower in correspondence to PF than CT (*p* < 0.001) and PO [*p* = 0.039; EM_UNS−PF_ = 1.35, 95% CI (0.94, 1.77); EM_UNS−CT_ = 1.95, 95% CI (1.54, 2.37); EM_UNS−PO_ = 1.81, 95% CI (1.39, 2.22)]. Figure 2a represents data concerning the two-way interaction and related statistically significant simple effects.

**Figure 3:**
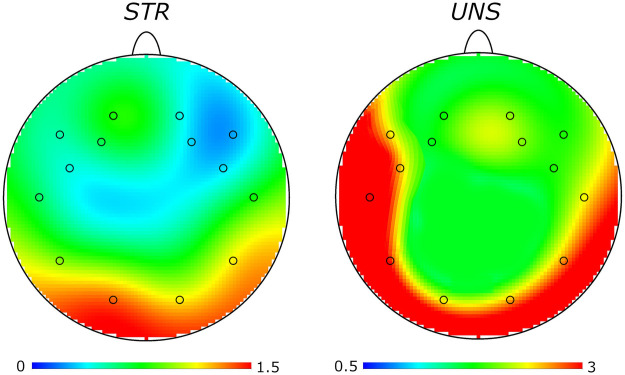
Topographical distributions of dyadic dissimilarity metrics (DDM) over a flat-head model in both structured (STR) and unstructured (UNS) interaction conditions. Blue-to-red colours mark progressively greater DDM values (i.e., lower Interpersonal Neural Synchrony, INS).

Again, pair-wise comparisons following the three-way interaction effect highlighted that DDM values were lower in the STR than the UNS condition specifically in correspondence of: right PF RoI [*p* < 0.001; EM_rPR−STR_ = 0.57, 95% CI (0.14, 1.00); EM_rPR−UNS_ = 1.45, 95% CI (0.97, 1.93)], right CT RoI [*p* < 0.001; EM_rCT−STR_ = 0.76, 95% CI (0.33, 1.19); EM_rCT−UNS_ = 1.57, 95% CI (1.09, 2.05)], left CT RoI [*p* < 0.001; EM_lCT−STR_ = 0.79, 95% CI (0.36, 1.22); EM_lCT−UNS_ = 2.34, 95% CI (1.86, 2.82)], and left PO RoI [*p* = 0.042; EM_lPO−STR_ = 1.23, 95% CI (0.80, 1.65); EM_lPO−UNS_ = 1.84, 95% CI (1.36, 2.31)]. Finally, in the STR condition, the right PF RoI showed lower DDM than the right PO RoI [*p* = 0.017; EM_rPF−STR_ = 0.57, 95% CI (0.14, 1.00); EM_rPO−STR_ = 1.18, 95% CI (0.75, 1.61)], while – in the UNS condition – lower DDM was found in left PF than left CT RoI [*p* < 0.001; EM_lPF−UNS_ = 1.26, 95% CI (0.78, 1.73); EM_lCT−UNS_ = 2.34, 95% CI (1.86, 2.82)], as well as in right then in left CT RoI [*p* = 0.017; EM_rCT−UNS_ = 1.57, 95% CI (1.09, 2.05); EM_lCT−UNS_ = 2.34, 95% CI (1.86, 2.82)]. Figure 2b represents data concerning the three-way interaction and related statistically significant simple effects. Figure 3 recreates the topographical distribution of DDM metrics over a flat-head model for both the STR and UNS conditions.

## 4 Discussion

This study aimed to deepen the understanding of communication dynamics between coach and athlete by investigating their neurofunctional correlates via a naturalistic hyperscanning paradigm. Specifically, we focused the analysis on EEG-derived INS metrics (Dyadic Dissimilarity Metrics, DDM) as markers of interpersonal neural similarity in activation patterns, specifically alpha-band power variations, during both structured and unstructured communication exchanges where feedback on training and competition performance was shared. As a secondary objective, we also wanted to explore the feasibility of an integrated neuroassessment protocol dedicated to the quality of Co-At communication and based on a naturalistic EEG hyperscanning in the sport context.

To our best knowledge, this is the first study to apply EEG hyperscanning within sport science to investigate interpersonal neural dynamics during real coach–athlete interactions in an ecological setting. By combining mobile EEG, naturalistic interaction contexts, and both subjective and neural measures, the present work extends two-person neuroscience approaches to a core applied sport domain that has so far remained largely unexplored from a neurophysiological perspective. At the same time, the findings should be interpreted within the limits inherent to naturalistic hyperscanning research. In line with contemporary accounts, interpersonal neural similarity is here considered an indirect, context-dependent marker of interpersonal attunement rather than a direct index of shared mental representations or identical cognitive states.

Taking into account our exploratory hypotheses, data analysis highlighted four main points: (i) during the structured feedback exchange communication was generally perceived as more effective than during unstructured ones, and athletes also perceived such opportunity for interaction as more affectively engaging; (ii) lower DDM (i.e., greater INS) metrics was generally observed during structured with respect to unstructured Co-At communication exchanges; (iii) DDM values was lower during structured than unstructured Co-At communication exchanges especially in correspondence of right pre-frontal, bilateral centro-temporal, and left parieto-occipital regions; and (iv) during the structured condition the right prefrontal regions exhibited lower DDM than the posterior ones, while during the unstructured communication condition the left centro-temporal regions showed the highest DDM – significantly greater than the neighbouring left prefrontal and right centro-temporal ones.

As for the first point, analysis of self-report data on the quality and perceived efficacy of Co-At interactions revealed that communicative exchanges in the structured condition were perceived as more effective than those in the unstructured one by both athletes and coaches. Such observation corroborates our first hypothesis. The implicit structure of a classical coaching-style interaction, the explicit management of turn-taking, and the greater degree of control over the relational milieu and emerging interpersonal dynamics have, plausibly, fostered athlete's and coach's capacity to attend to verbal and non-verbal information, to focus on the communicative goals, as well as to share and better specify meanings, thoughts, and sensations, thus resulting in a greater perception of efficacy in conveying them (65). Also, the protection from external nuisance or intrusions and the structured format facilitate a bidirectional interaction and help the division of labour for communicative success (66). This have likely provided clear, step-by-step feedback and enabled athletes and coaches to absorb shared thoughts and experiences, as well as strategies and performance cues, more effectively and without the cognitive load that may arise from ambiguous or improvised feedback.

In addition, the analysis of self-report data unexpectedly highlighted that athletes have felt more affectively engaged during the structured interaction condition than during the unstructured one. While this might seem illogical given that the unstructured exchanges occurred during an emotion-laden and challenging situation (i.e., a match), we suggest that it might actually be due to the affective load of the competitive context, which might have – by difference – hampered the perception of affective engagement specifically for communication exchanges with the coach. Alternatively (or perhaps complementarily), the high reported affective engagement of athletes during the structured interaction with the coach might follow induced or re-evoked emotional responses when talking about previous competitions, training, and related subjective experience.

As for the second main point, lower DDM metrics was generally observed during structured with respect to unstructured Co-At communication exchanges. Such finding, besides deposing in favour of our second main hypothesis, is also consistent with self-reported perception of efficacy in communication during the structured condition. Also, it aligns with Stephens et al. (38), who found that synchronized brain activity among participants correlated with effective communication. In structured conditions, the predictability and clarity of the interaction likely facilitate alignment in neural activation. It is, indeed, plausible that the implicit nature of structured communicative interactions facilitates coordination between inter-agents, the creation of shared meanings, and co-regulation of information flows, fostering interpersonal neural similarity. And again, organized flow of information might foster joint attentional co-regulation, further strengthening INS (40, 43, 51).

Furthermore, alpha oscillations are theorized to reflect active inhibition and top-down regulation mechanisms that allow filtering out task-irrelevant information and modulate resources on a task (54, 55, 67). Also, consistently, modulation of alpha has been associated with the activity of the mirror neuron system, suggesting potential association which imitation and empathy-related processes crucial for effective social understanding and coordination (44, 46, 47, 49, 50). Given such premises, we suggest that the fact that inter-agents neurofunctional responses in such frequency range become more similar during the structured interaction might mirror greater achieved attunement and a consistent use of mental resources on social understanding, regulation and coordination processes. Within a structured context – where the information flow is well-defined and more predictably organized – both coach and athlete benefit from a shared focus on the communicative task, which plausibly supports alignment of their attentional resources and facilitates neural similarity in relevant brain areas. Also, the implicit and predictable organization of the structured interaction condition, the naturally emerging bidirectionality of communication exchanges, and the actual opportunities to share complex narrations concerning own training/competition experience might have provided greater room for building empathic resonance, defining a common representational ground, and developing relational closeness.

In contrast, unstructured feedback conditions displayed higher DDM values, suggesting greater dissimilarity in alpha activation patterns between coach and athlete. This increased neural dissimilarity may stem from the more spontaneous and less predictable nature of the interaction, which could limit INS. Indeed, unstructured interaction situations – such as, in our case, real-time Co-At exchanges during a match – occur and develop dynamically, fluidly following and adapting to unpredictable cues and constraints that connote the specific environment and moment in which the interaction takes place. This might hinder intuitive anticipation and adaptation of inter-agents to each other communication and social cues, undermining joint attention and syntonization processes that foster successful inter-action (31, 40, 41).

Notwithstanding the remarkable motivational-affective salience of the competitive situation and the shared goal, with both athlete and coach aiming at success in the match, each member of the dyad might focus on different aspects of the interaction. The athlete may be primarily concerned with immediate performance, understanding of coaches suggestions, and adapting to feedback, while the coach may primarily focus on strategic planning of communication to make it as clear and impactful as possible given situational constraints, as well as on using his/her real-time feedback to help the athlete regulate his/her performance and mental state. This may plausibly lead to greater divergence in neural activation patterns, mirroring partly independent regulation of their focus and processing demands.

These findings are consistent with theories suggesting that INS varies according to task structure and clarity of goals (8, 31, 40, 68). The structured condition, by promoting greater predictability and coordination of communicative intent and attentional focus, may be associated to more aligned and similar activation patterns and cognitive engagement between the dyad, reflected in reduced DDM. Conversely, the variability in the unstructured condition may lead to more individualized embodied experience in terms of sensations, cognitions, and emotions, where both coach and athlete prioritize different aspects of the communication exchange as well as of related affective and sensorimotor connotations. This setting allows for flexibility, but at the potential cost of reduced neural similarity – as mirrored by the higher DDM values – plausibly due to greater attention to idiosyncrasies in the interaction experience – as suggested by different perception of affective engagement in athletes/coaches self-reported data.

The interpretation above is further supported and better specified by the emerging evidence related to the specific topographies of higher/lower INS in the structured vs. unstructured conditions (i.e., the third and fourth main discussion point). Indeed, greater INS (as marked by lower DDM values) during the structured vs. unstructured was specifically found in right pre-frontal, bilateral centro-temporal, and left parieto-occipital regions, suggesting the involvement of a quite defined set of cortical regions. And again, while, in the structured interaction condition, the highest INS was observed in right prefrontal regions, in the unstructured one the least INS was observed in left centro-temporal regions, specifically with respect to the neighbouring left prefrontal and right centro-temporal ones.

The regional specificity of alpha activity, particularly in frontal and parieto-occipital areas, reflects the brain's capacity for top-down regulation of cognitive processes and behaviour, and attentional control. Present findings are consistent with observations by Kelsen et al. (69), who found that during communication exchanges, prefrontal regions display intense activation, indicating increased load on working memory, attention, and concentration. Similarly, parietal and temporal regions – key for auditory processing and social interaction – show heightened activity. This likely occurs because, as Kawasaki et al. (52) suggested, during verbal interactions characterized by turn-taking, inter-agents must not only focus on the content and context of the conversation but also coordinate the duration, intervals, rhythm, and timing of the interaction itself.

Moreover, as noted above, alpha activity has been associated with the activity of the mirror neuron system, thought to support imitation, empathic resonance, sensorimotor simulation/mirroring, and social understanding (44, 46, 47, 49, 50). Consistent with Kelsen et al. (69), our study found that similar responses are particularly pronounced in the prefrontal and centro-temporal regions during structured conditions. This result is significant, as these areas are crucial for mentalization and perspective-taking processes in social interactions (19, 70). Mentalization processes are essential for anticipating and interpreting communicative signals and the intentions of others, supporting self-orientation and contributing to effective communication (69, 71, 72). Interestingly, we observed stronger right-lateralized prefrontal INS in the structured condition, consistent with the role of the right hemisphere in processing and regulating affects, integrating salient emotional cues, and facilitating related non-verbal forms of communication (28, 73–75). Such interaction condition, indeed, have likely facilitated accessibility to non-verbal, besides verbal, contents for both inter-agents, who were seated next to each other in a relatively isolated setting. The ease of access to a richer set of social cues might have mirrored in a greater and more consistent involvement of inter-agents' prefrontal structures supporting and regulating to social perception, understanding, and coordination processes.

In contrast, low levels of inter-brain similarity were observed in the unstructured condition, especially in the left centro-temporal region. Although the prefrontal region exhibited slightly better alignment than other areas, these levels remained lower than those seen in structured conditions. This finding is consistent with Koul and colleagues’ (58) assertion that attunement between individuals can occur even in the absence of structured tasks. Indeed, during communicative exchanges, the alignment of brain activation profiles extends beyond the vocal-auditory domain of speech, establishing joint attention necessary for effective mutual understanding (76, 77). To sum up, present findings suggest that, even in the unstructured condition, the two brains achieved a certain degree of overlap in activation patterns, suggesting that communicative interaction can foster joint attention and mutual understanding even without a predefined or expectable structure in communication exchanges. However, it still has to be underlined that, in the highly dynamic and complex context of unstructured Co-At interactions, communication has likely been remarkably influenced by situational factors (78–80). Such factors, combined with the absence of systematic bidirectional interactions, have likely contributed to lower INS between coach and athlete.

Furthermore, in unstructured interactions, the sensorimotor and perceptual experiences of the athlete and coach are highly individualized, whereas in structured interactions, these experiences may be more closely aligned. As noted above, while still sharing common medium-term goals – i.e., to perform at the best of actual possibilities and, hopefully, to win the match – athletes and coaches involved in unstructured feedback exchanges in the context of a match experience such social interaction from very different perspectives in terms of sensations, proximal intentions, thoughts, and emotions. Their mindset, their situated processing of internal and external information, their embodied experiences plausibly presents smaller overlapping areas. This divergence may explain the lower similarity observed, especially, in left centro-temporal more than the left pre-frontal and right-centro-temporal regions in unstructured conditions. The finding that INS in the unstructured condition was still higher in the prefrontal regions than the posterior ones suggests, indeed, that -despite differences in sensorimotor and perceptual experiences – the activation of regions involved in self-regulation and higher socio-cognitive functions (19, 70) remain aligned. At the same time, the increased dissimilarity in central-temporal and parieto-occipital regions may specifically reflect the specificities in sensorimotor and perceptual experiences inherent to the coach and athlete during the unstructured feedback conditions (57), which occurred within an actual match context during which the athlete and the coach had to assume different stances and perspectives.

As a final point, it is relevant to note that the present pilot exploratory study presents strengths and limitations that warrant consideration for a full appreciation of potential implications of the findings. Among the primary strengths is the innovative use of the hyperscanning paradigm in a completely naturalistic context, investigating real-life complex interactions between athletes and coaches. That advanced paradigm allows for simultaneous recording of brain activity in multiple individuals during social interactions, providing insights into the neurophysiological dynamics underpinning Co-At interactions. Additionally, the ecological validity of the study stands as another strength, as data were collected in real, dynamic contexts representative of athletes' and coaches' everyday experiences. This authentic setting offers a practical and realistic view of Co-At interactions, ensuring that the findings are both applicable and relevant. Another strength of the study lies in the integrated use of both electrophysiological measures of INS with self-report questionnaires, thus complementing objective data on neural activation with subjective perspectives on interaction quality.

Yet, several limitations of the present study should be acknowledged. First, the relatively small sample size reflects the exploratory nature of the work and the practical constraints inherent to collecting hyperscanning data in ecological sport settings involving real coach–athlete dyads. While this limits the generalizability of the findings, it is consistent with current naturalistic hyperscanning research and was partially mitigated through the use of mixed-effects modelling and within-dyad analyses. Second, while the absence of experimental control conditions and the reliance on real-world interaction contexts were deliberate methodological choices aimed at maximizing ecological validity, the observed interpersonal neural synchrony should be cautiously interpreted in relation to naturally unfolding communicative dynamics rather than as sole evidence of determined causal mechanisms. Also, the focus on a single sport discipline – though functional as a starting point for applied research – may not adequately represent the broader population of athletes and coaches. Another significant limitation intrinsically lies in potential constraints of the naturalistic setting used for data collection. Although collecting data in real-world contexts enhances ecological validity and practical relevance, it might also introduce situational and environmental confounding variables that have to be taken into account and controlled as much as possible by balancing respect of natural interaction modalities with strict procedural control during data collection and methodological precaution in data processing and analysis. And again, while interaction segments considered for analysis were carefully matched and restricted to stationary periods across conditions, residual contextual differences related to competitive pressure and arousal may still contribute to the observed effects and cannot be entirely ruled out in a naturalistic design. Finally, while multiple procedural and preprocessing steps were implemented to minimize the impact of movement-related artefacts, it cannot be entirely eliminated in naturalistic EEG recordings collected in real-life settings or during sport activities. Residual noise is therefore an unavoidable feature of the dataset and should be considered when interpreting the results, reinforcing the view of INS as an indirect and context-sensitive marker rather than a precise measure of shared neural processing.

## 5 Conclusions

This study provides initial evidence that EEG hyperscanning can be meaningfully applied within sport science to investigate the neurofunctional correlates of real coach-athlete communication. By capturing interpersonal neural similarity in naturalistic training- and competition-related contexts, the present work suggest new methodological and conceptual avenues for studying relational processes that are central to athletic development and performance.

The multifaceted nature of Co-At communication brings both opportunities and challenges. Effective communication can significantly improve learning outcomes, foster resilience, and increase the athlete's intrinsic motivation, all while optimizing performance. Our findings underscore the emergence of neurofunctional correlates of interpersonal syntonization during communication and feedback exchanges between coaches and athletes in different naturalistic interactions. They hint at the importance of working to deliver feedback efficiently before, during, and after a competition. By fostering a state of syntonization, coaches may enhance the athlete's receptiveness to guidance, potentially improving the impact and usefulness of feedback. This highlights the value of structuring feedback in a way that is clear, adaptive, and contextually relevant, supporting both concurrent performance adjustments and prospective athlete progresses. Conversely, poorly timed or overly directive feedback may hinder learning by creating dependence or reducing autonomy (81). Furthermore, the physical and emotional demands of high-performance environments often pose challenges in maintaining consistent, empathic communication, especially under conditions of stress or fatigue. Coaches must navigate these complexities while balancing short-term performance goals with the athlete's longer-term development.

The emerging field of two-person neuroscience provides a valuable framework for understanding the neurophysiological basis of interpersonal communication even in the context of competitive sports, providing novel insights for neuroassessment practice (82, 83). This approach moves beyond the traditional study of isolated brain activity to examine the bidirectional and dynamic exchanges that occur between individuals during social interactions. For the sake of clarity, we acknowledge that interpersonal neural similarity should be interpreted as an interaction-dependent marker whose functional meaning emerges only in relation to task structure, communicative demands, and contextual constraints, rather than as a direct proxy for shared cognition or experiences. Given such constraints, present findings, by looking at real-life communication exchanges in naturalistic contexts, suggest that the Co-At relationship may operates as a joint system, where the inter-agents' neural activities may show overlapping patterns even in challenging situations and unstructured contexts – such as during a match.

## Data Availability

The raw data supporting the conclusions of this article will be made available by the authors, without undue reservation.
